# Supplementary material for: NMI and IFP35 serve as proinflammatory DAMPs during cellular infection and injury
Source: Nat Commun. 2017 Oct 16;8:950. doi: 10.1038/s41467-017-00930-9 (PMC5643540; doi:10.1038/s41467-017-00930-9)
Supplement: Supplementary file 1 — Supplementary Information [file 41467_2017_930_MOESM1_ESM.pdf]

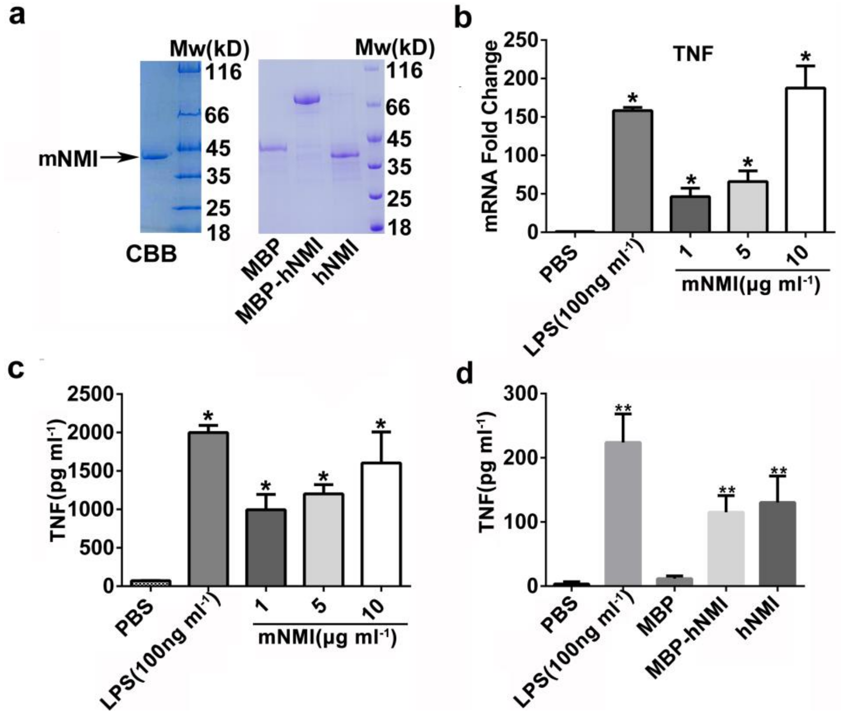

Supplementary Figure 1. Recombinant NMI induces cytokine release in macrophages. (a) SDS-PAGE analysis of purified mNMI protein (1 µg) and hNMI protein (1 µg) stained with Coomassie blue. (b) Tumor necrosis factor (TNF) mRNA abundance in mouse bone marrow-derived macrophage (BMDM) cells stimulated with increasing concentrations of recombinant mNMI or 100 ng ml^{-1} lipopolysaccharides (LPS) for 4 hours, as detected by RT-PCR using glyceraldehyde-3-phosphate dehydrogenase as internal controls. (c) TNF production in BMDM cells stimulated with increasing concentrations of recombinant mNMI or 100 ng ml^{-1} LPS for 4 hours. (d) TNF level in the supernatants of THP1 cells was analyzed by enzyme-linked immunosorbent assay (ELISA) 8 hours post incubation with LPS (100 ng ml^{-1}) and different purified proteins (5 µg ml^{-1}). Error bars in panel b, c and d indicate ± s.e.m. from 3 independent experiments. Significance was tested by one-way ANOVA followed by Student-Newman-Keuls test. *P<0.05, **P<0.01 compared with PBS control.

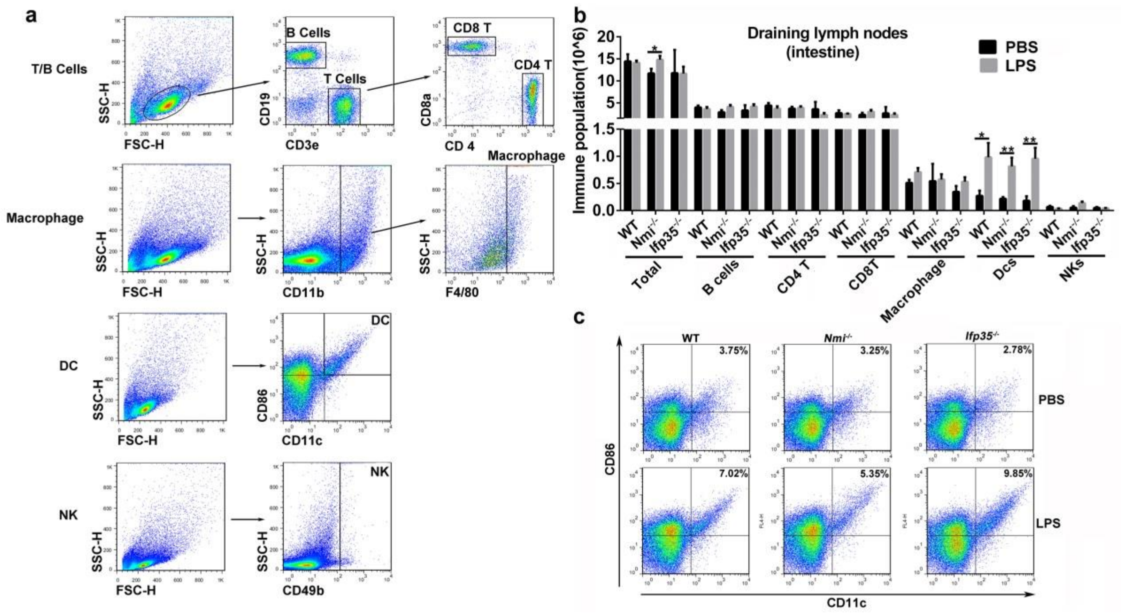

Supplementary Figure 2. The LPS induced septic-shock mice model. (a) Gating strategies to determine the overall percentage of each immune populations that migrate into mesenteric lymph nodes, including B cells, CD8 T cells, CD4 T cells, macrophages, DCs and NKs. (b) Mobilization of immune populations to lymph nodes after injection of LPS (10mg kg^{-1}) into wild type and knockout C57BL/6 mice. Data are presented as the mean ± s.e.m. of 3 individual mice. Significance was tested by unpaired student's t-test. *P<0.05, **P<0.01. (c) Flow cytometric analysis and quantification of $CD11c^{+}CD86^{+}$ cell populations in lymph nodes.

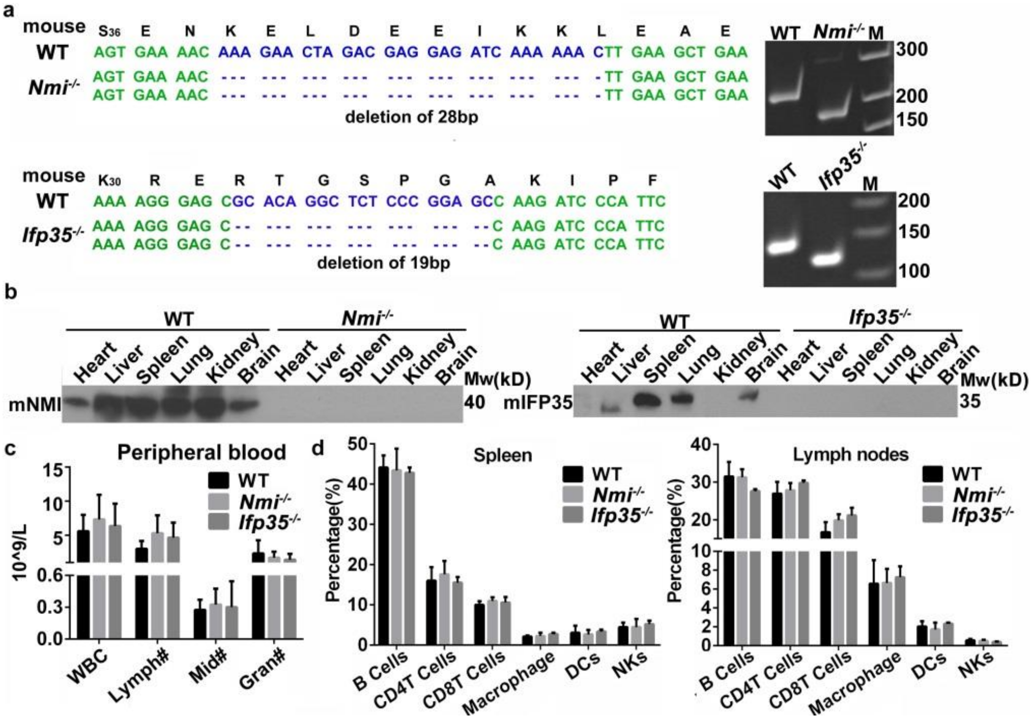

Supplementary Figure 3. Generation and characterization of *Nmi* and *Ifp35* deficient mice. (a) Generation of *Nmi* and *Ifp35* deficient mice using CRISPR-Cas9 technology. The deletion and frame shift of the *Nmi*$^{-/-}$ and *Ifp35*$^{-/-}$ mice genomes are shown. (b) Western blot analysis of NMI and IFP35 in different organs of WT, *Nmi*$^{-/-}$ and *Ifp35*$^{-/-}$ mice. (c) Comparative quantification of cell populations from the peripheral blood. Data are presented as the mean ± s.e.m. of 5 individual mice. (d) Percentage of the immune cells isolated from spleen and lymph nodes. Data are presented as the mean ± s.e.m. of 3 individual mice.

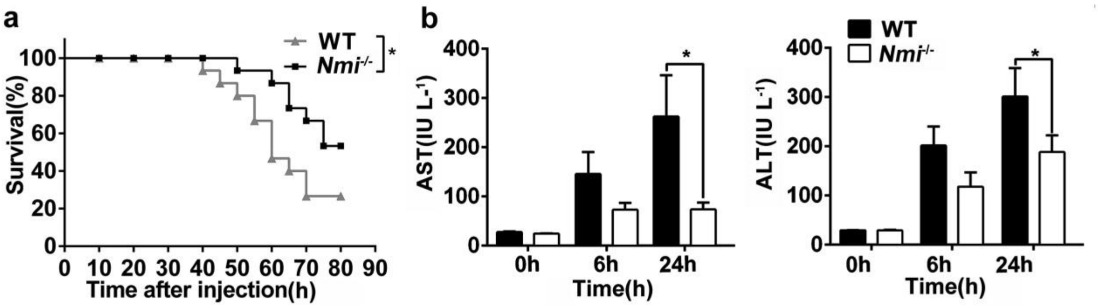

Supplementary Figure 4. NMI knockout mice attenuate inflammatory responses. (a) The survival rate of wild type (n=15) and *Nmi*$^{-/-}$ mice (n=15) intraperitoneally injected with 1×10^4 live *S. typhimurium.* The figure shows a summary of the survival rates of both groups over 80 hours. Significance was tested by log-rank test. *P<0.05 compared to WT. (b) Serum concentrations of aspartate transaminase (AST) and alanine transaminase （ALT）in *Nmi*$^{-/-}$ and WT mice as determined by ELISA 6 or 24 hours after injection of acetaminophen (APAP, 600 mg kg^{-1}). Data are presented as the mean ±s.e.m. of 5 individual mice. Significance was tested by unpaired student's t-test. *P<0.05.

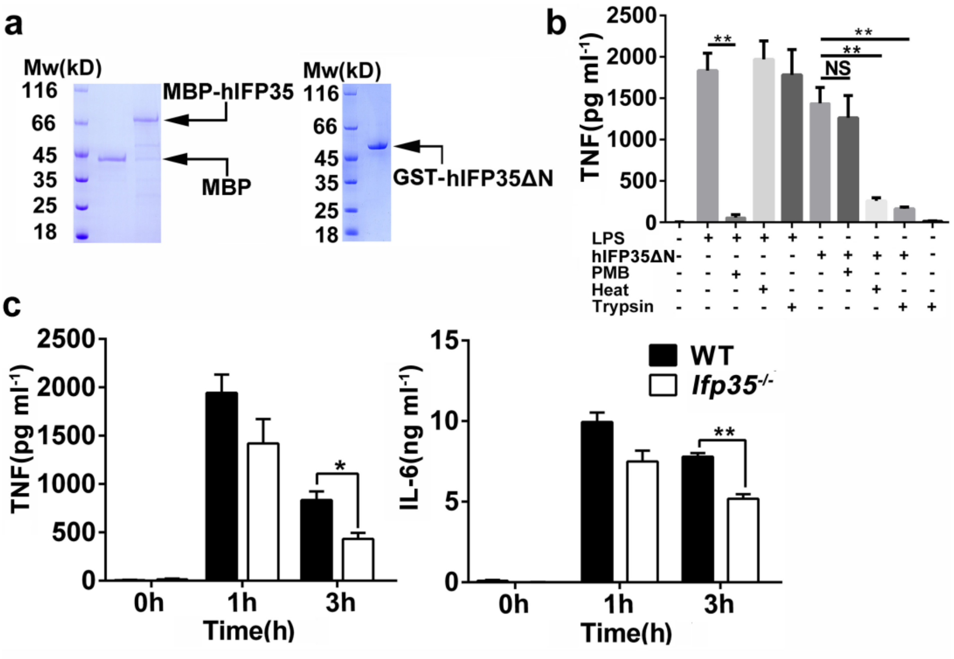

Supplementary Figure 5. IFP35 serves as DAMP and stimulates the inflammatory responses. (a) SDS-PAGE analysis of purified hIFP35 with maltose-binding protein (MBP) tag and Glutathione S-transferase (GST) tag, MBP-hIFP35 (1 μg) and GST-hIFP35ΔN protein (1 μg) stained with Coomassie blue. (b) TNF production in RAW264.7 cells stimulated with hIFP35ΔN (5 μg ml^{-1}) or LPS (100 ng ml^{-1}) for 8 h with (+) or without (−) pretreatment of polymyxin B (PMB; 25 μg ml^{-1}), heat (80 ℃ for 30min) and Trypsin. Data are presented as the mean ± s.e.m. n = 3 independent experiments. Significance was tested by one-way ANOVA followed by Student-Newman-Keuls test. *P<0.05. (c) TNF and IL-6 in the sera of *Ifp35*$^{-/-}$ and WT mice as determined by ELISA 1 hour or 3 hours after injection of LPS (50 μg kg^{-1}) and D-gal (1g kg^{-1}). Data are presented as the mean ± s.e.m. of 5 individual mice. Significance was tested by unpaired student's t-test. *P<0.05, **P<0.01.

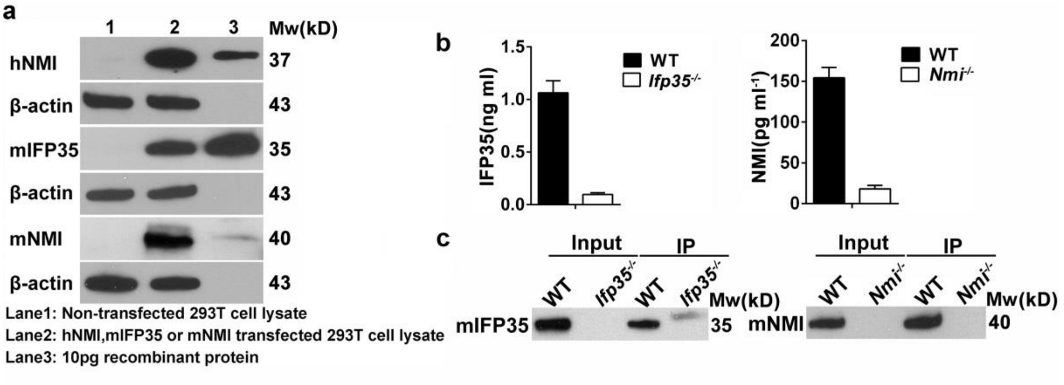

Supplementary Figure 6. The specificity of the antibodies and ELISA kits used in this paper. (a). HEK293T cells (ATCC CRL -11268™) were transfected with hNMI, mIFP35 or mNMI and the specificity of anti-hNMI (ab183724), anti-hIFP35 (H00003430-D01P, also used as antibody for mIFP35) and anti-mNMI were assessed by immunoblotting. (b-c) The specificity of the ELISA kits. Panel b shows the ELISA analysis of IFP35 and NMI in the serum of WT, *Nmi*$^{-/-}$ or *Ifp35*$^{-/-}$ mice, treated with LPS (10 mg kg^{-1}). Panel c shows the proteins bound to antibody in the ELISA kits, assessed by immunoblotting. The WT, *Nmi*$^{-/-}$ or *Ifp35*$^{-/-}$ BMDM cells were washed twice with cold PBS and collected in lysis buffer. The whole cell lysates were incubated at 4 ℃ for 45 min, followed by centrifugation (12 000 ×g for 15 min, at 4 ℃). mIFP35 or mNMI antibody from the ELISA kits was coupled with Protein G beads. The beads with antibody were incubated with cell lysates at 4 ℃ for 4 hours and washed 4 times with lysis buffer. Proteins bound to the beads were separated by SDS-PAGE and immunoblotted with anti-hIFP35 (H00003430-D01P) or anti-mNMI.

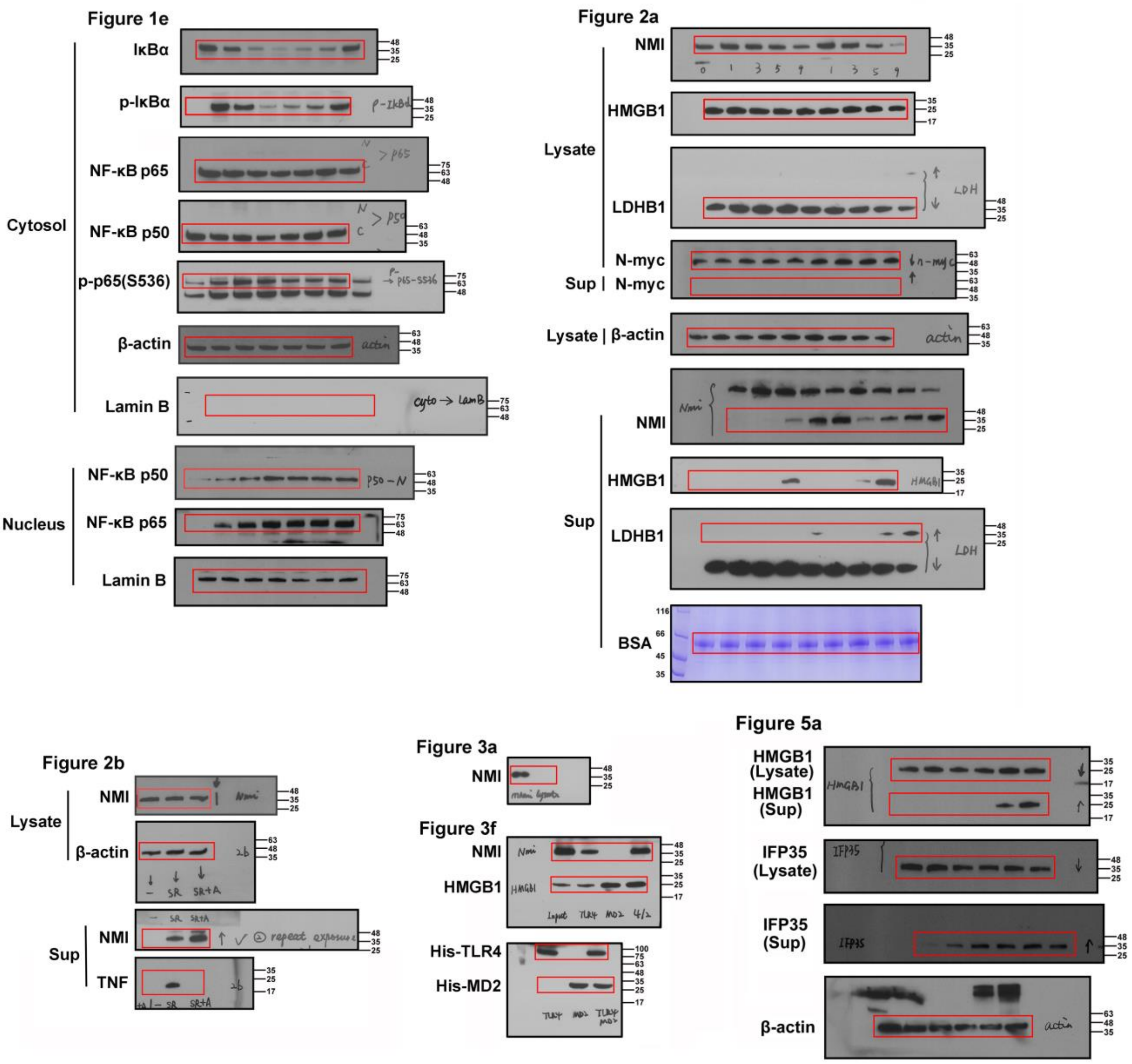

Supplementary Figure 7. Original data of immunoblots corresponding to indicated figures.

Supplementary Table 1 NMI in the sera of patients with infection.

Patients	NMI (pg ml^{-1})	Clinical manifestation	After treatment
X1	55.55	Severe pneumonia, sepsis	recovered
X2	76.8	Abdominal infection, sepsis	recovered
X3	3825	Acute diffuse peritonitis	deceased
X4	5770	Sepsis	deceased
X5	23.1	Diffuse peritonitis	recovered
X6	13.35	Sepsis	recovered
X7	67.6	Acute suppurative cholangitis, sepsis	recovered
X8	28.4	Sepsis	recovered
X9	132.26	Severe pneumonia, sepsis	recovered
X10	122.65	Urinary infection,　bacteremia	recovered
X11	33.38	Cholangitis,　sepsis	recovered
X12	2321.23	Severe pneumonia, bacteremia	recovered
X13	543.48	Severe pneumonia, perianal infection	recovered
X14	358.87	Sepsis	recovered
X15	1184.94	Sepsis	deceased
X16	119.45	Abdominal infection	recovered
X17	74.73	Severe pneumonia, sepsis	recovered
X18	109.85	Infective endocarditis	recovered
X19	300.15	Deep incisional surgical site infection	recovered
X20	103.46	Bacteremia	recovered
X21	25.44	Infective endocarditis	recovered
X22	314.80	Severe pneumonia, bacteremia	recovered
X23	817.46	Pneumonia, duodenal ulcer，bacteremia	deceased
X24	398.18	Urinary infection, bacteremia	recovered
